# Professor Thoma Ionescu (1860–1926) founder of the Modern Romanian School of surgery

**Published:** 2010-02-25

**Authors:** F Popa, O Buda, LV Purcarea

**Affiliations:** ‘Carol Davila’ University of Medicine and Pharmacy, Bucharest Romania

The year 2010 will be proclaimed the ‘Thoma Ionescu Year’ for the Romanian medicine. This occasion marks a unique double anniversary: 150 years since the birth of the great scientist and 100 years since his surgical demonstrations during an Anglo–American tour in New York, Philadelphia, Rochester and Chicago.

Brother of politician Take Ionescu, Thoma signed up for the University of Medicine in Paris, in 1879, after graduating ‘Sfantul Sava’ High School, while, at the same time, attending the courses of the Faculty of Law, which he graduated in 1882. After this, he passed the exams for non–resident and resident medical studentship, thus completing his clinical and surgical training.

Simultaneously with his activity in the hospital as an extern and an intern, he gained the titles of associate anatomy prosector in 1887, and afterwards full anatomy prosector at the Faculty of Medicine in Paris, after passing certain examinations where he got the first prize.

In 1885, after another examination, he was appointed provisional intern of the hospitals and worked at Bicetre, in Bourneville's Epilepsy Department. In 1886, he was appointed full intern of the hospitals and he spent all four years in surgery, being an intern for Peyrot (Bicetre), Paul Berger (Tenon), Le Dentu (St. Louis) and professor Verneuil (Pitie–Salpetriere). In 1890, he competed for the medal offered to the interns of the 4^th^ year, for the Surgery Department, and he obtained the second place, thus receiving the silver medal in Surgery. In the same year, his memorial, presented at the competition for the medal, and entitled Internal Retroperitoneal Hernias, obtained the ‘Laborie’ prize of the Academy of Medicine in Paris, a prize worth 5 000 francs, awarded to ‘the work which determined the faster progress in Surgery in the current year’. Together with being granted with this prize, the physician Thoma Ionescu became laureate of the Academy of Medicine in Paris.

In 1892, he presented his PhD thesis, entitled *L'evolution intrauterine du colon pelvien / Intrauterine Evolution of the Pelvic Colon*, and, after an exam where he won the first prize again, Thoma Ionescu obtained the title of associate professor in Paris (1892–1894). In the same year, the Ministry of Public Education in Paris commissioned Thoma Ionescu, who was at that time prosector in Paris, to study and report the anatomy teaching methods used at the universities of Germany and Austria.

In 1894, he wrote a chapter entitled *Anatomie du tube digestif / Anatomy of the Digestive Tract*, in the important anatomy treaty elaborated by Poirier and Charpy, where he described for the first time in the medical literature, the duodeno–jejunal fossae, the sigmoid fossae, the rectal sheath and the rectal wings.

In 1895, due to a special law, the Department of Topographical Anatomy and surgical clinic was founded in the Faculty of Medicine in Bucharest, and, Thoma Ionescu was asked to become the chairman. In 1897, he participated in the International Medical Congress in Moscow, where he presented the resection of the cervical sympathetic in exophthalmic goiter and epilepsy.

At the 1898 Marseilles Congress of Gynaecology, obstetrics and paediatrics, Professor Thoma Ionescu gave a lecture on the complete abdominal castration in case of uterus– annex septic lesions. Anatomist by formation, then surgeon, he understood from the beginning that the fundamental bases of the surgical education also rely on the knowledge of the surgical anatomy besides the experimental surgery. That is why the Institute of Anatomy was divided by Ionescu into a Department of Topographical Anatomy and one of Experimental Surgery. The knowledge of topographical and operational anatomy allowed both the teacher and his school to imagine or improve a number of surgical techniques and methods, some of which are also valid today (for instance the technique of Thoma Ionescu type large hysterectomy, the technique of cervical sympathectomy, etc.).

His other contributions, considered classic, regard the anatomical study of the pharynx, peritoneum, abdomen, duodenum, pelvic colon, etc. (*Anatomie topographique du duodenum et hernies duodenales / Topographical Anatomy of the Duodenum and Duodenal Hernias *). He innovated numerous fields of surgery, practicing the extraction of tumours of the optic nerve without damaging the globe of the eye; he conceived and accomplished a radical operation of uterine cancer and of the suppurations of the annex glands; he introduced new surgical procedures in splenectomy, by removing the spleen; in the surgery of the duodenal ulcer, the rachianesthesia for abdominal surgery, the resection of the rectum; the resection of the stomach in case of severe ulcers; he introduced a special procedure in the temporal–maxillary ankylosis; in herniorrhaphy, in craniotomy, by cutting a section of the skull for brain operations, etc. As a morphologist, he published important studies regarding the topographical anatomy of the pyloric region of the duodenum, of the pelvic colon. He presented the results of his researches in numerous scientific works, among which: *Tuberculose herniare / Hernial Tuberculosis* (1891); *Gastrectomies pour cancer / Gastrectomies for Cancer* (1891); *Technique operatoire des gastrectomies pour cancer / The Surgical Procedure of  the Gastrectomies for Cancer* (1891); *Sur la resection du nerf maxillaire et du ganglion de Meckel / On the Resection of the Maxillary Nerve and of Meckel's ganglion* (1896); *Resection totale et bilaterale du sympathique cervical dans le traitement du goitre exophtalmique et de l'epilepsie / Complete and Bilateral Resection of the Cervical Sympathetic in Curing Exophthalmic Goiter and Epilepsy* (1897);* Nouveaux procedes pour la cure radicale des henies inguinales / New Procedures for the Radical Cure of the Inguinal Hernias *(1897); *Temporary Craniotomy* (1898);* Treating Glaucoma by Resecting the Cervical Sympathetic* (1898); *Preliminary Cystotomy in the Resection of  the Urethra* (1898); *La splenectomie / The Splenectomy* (1898); *Traitement chirurgical du cancer de l'uterus / Surgical Treatment in the Uterine Cancer* (1900) and so on and so forth. He was also an innovator in the field of Anaesthesiology, promoting high class rachianesthesia, used in surgical procedures of the head and the neck, procedure known as the ‘Romanian method’ (*La Rachianesthesie generale / The General Rachianesthesia*, 1919); he also conceived a high number of surgical instruments, many of them for the use in the abdominal cavity.

In 1905, at the International Surgery Congress in Bruxelles, Thoma Ionescu presented, for the first time in the world, the principles of gastric resection in the non– cancerous diseases of the stomach (partial gastric resections).

Between 1909 and 1910, in New York and afterwards in Rochester, in brothers' Mayo clinic, Ionescu did surgical demonstrations on upper segments of the body, under high rachianesthesia. In the field of gynaecology, he described the procedure of Cuneo'hysterectomy for the correction of the retro deviations of the uterus and was one of the first in the world to accomplish the resection of the sacral sympathetic in pelvic pains, by using his own technique.

In 1902, while dealing with the cancer of the cervix, he proved, on the basis of the histological examinations of pelvic ganglions, that the Wertheim operation is incomplete and he recommended the extended lymphadenectomy of the pelvis. Thoma Ionescu is one of the first surgeons in the world preoccupied with the surgery of the sympathetic. He extended the exeresis of the cervical sympathetic chain to the first thoracic ganglion in Basedow disease and in epilepsy, before Jaboulay. He extended the indication of this kind of intervention in glaucoma, essential migraine and angina pectoris, and associated stellectomy with the cervical–thoracic sympathectomy (in 1916, together with Victor Gomoiu). These studies are included in *Le sympathique cervico–thoracique / The Cervico–thoracic Sympathetic *(Masson Publishing House, Paris, 1923) monograph.

He founded the journals ‘Archives des Sciences Medicales’ (1896) and ‘Revue de Chirurgie’ (1897), being also the founder and organizer, together with Constantin Dumitrescu Severeanu, of the Surgery Society in Bucharest, in 1898.

Thoma Ionescu was elected three times Dean of the Faculty of Medicine in Bucharest, between 1906–1912, 1921–1923, and 1925–1926. Between 1912 and 1915 he was Rector of the University of Bucharest.

He was one of the supporters of the creation of the Romanian national state: in 1918 he took part in the National Council of Unity of the Romanians in Paris. He was the first Romanian delegate to the sessions of the United Nations, between 1920 and 1921.

In June 1925 he became a member of honour of the Romanian Academy.

